# An efficient method to improve the quality of tetrahedron mesh with MFRC

**DOI:** 10.1038/s41598-021-02187-1

**Published:** 2021-11-23

**Authors:** Yuzheng Ma, Monan Wang

**Affiliations:** School of Mechanical and Power Engineering, Harbin University of Science and Technology, Harbin, China

**Keywords:** Engineering, Mathematics and computing

## Abstract

In this paper, we proposed a novel operation to reconstruction tetrahedrons within a certain region, which we call MFRC (Multi-face reconstruction). During the existing tetrahedral mesh improvement methods, the flip operation is one of the very important components. However, due to the limited area affected by the flip, the improvement of the mesh quality by the flip operation is also very limited. The proposed MFRC algorithm solves this problem. MFRC can reconstruct the local mesh in a larger range and can find the optimal tetrahedron division in the target area within acceptable time complexity. Therefore, based on the MFRC algorithm, we combined other operations including smoothing, edge removal, face removal, and vertex insertion/deletion to develop an effective mesh quality improvement method. Numerical experiments of dozens of meshes show that the algorithm can effectively improve the low-quality elements in the tetrahedral mesh, and can effectively reduce the running time, which has important significance for the quality improvement of large-scale mesh.

## Introduction

The finite element method has a wide range of applications in scientific computing and engineering problems. When solving problems with the finite element method, the solution domain needs to be decomposed into a set of finite simple elements. The rapid development of mesh generation technology provides a strong guarantee for the application of finite element methods for numerical simulation^1–3^. However, mesh generation is only the beginning, the quality of the generated mesh often determines the accuracy of the finite element calculation. In many cases, the quality of a mesh cannot meet the requirements. Therefore, it is indispensable to improve the quality of the mesh through mesh improvement to meet the requirements. The quality of the mesh depends on the worst element. If there are too many low-quality elements in the mesh, the solution error will increase, and the solution time will become longer or even cannot be calculated^4–8^. For example, in the virtual surgery system, cutting is the basic operation. Virtual cutting of the soft tissue model changes the topology of the mesh model. In the process, the mesh model needs to be refined. Therefore, soft tissue cutting will produce some low-quality tetrahedrons^9–13^, which can result in deformation errors or reduced computational efficiency. Thus, mesh quality improvement plays an important role in soft tissue cutting simulation.

Commonly used operations in existing mesh improvement methods include: (1) mesh smoothing. The smoothing operation improves the mesh quality by adjusting the vertex positions; (2) topology operation. The topological operations improve the mesh quality by changing the connections of vertices in the target area; (3) vertex insertion/deletion. Vertex insertion/deletion improves mesh quality by inserting new vertices into the mesh or deleting existing vertices in the mesh.

**Figure 1:**
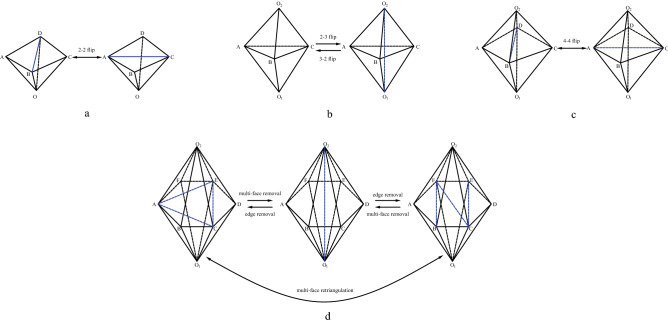
Topology operations.

In this paper, we focus on improving mesh quality through topological operations. As mentioned above, the topology operation improves the mesh quality by changing the connection mode of the vertices in the target area. The problems that the topology operation needs to solve are two problems:(1) how to obtain the optimal connection method of the target area; (2) how to complete the operation within an acceptable running time. The most commonly used topological operation is the mesh flip^14^, including 2-3, 3-2, 4-4flip (Fig. 1) (m-n flip here means replacing the original m elements with n elements). These operations are collectively called basic flips, and these types of mesh flips can only handle a few tetrahedral elements at a time. Based on these basic flips, researchers have proposed several advanced flip operations, including edge removal^15^, multi-face removal^16^, multi-face retriangulation^17^ (Fig. 1), which improved the limitation of the limited number of processing elements. Advanced flips can be implemented by iteratively performing basic flips, where edge removal and multi-face removal algorithms are implemented in Stellar^18^. Performing topological operations in a larger area means that there are more ways to connect vertices, and more ways to connect vertices means that there is a greater possibility to improve the quality of the elements in the target area to a higher level^19^. Improving mesh quality through mesh reconstruction is a valuable research direction.

The main contribution of this research is to propose a topology operation algorithm for local mesh reconstruction, MFRC (Multi-face reconstruction), and develop a tetrahedral mesh improvement method based on MFRC combined with smoothing operations. Although multi-face removal improves the mesh quality more effectively than basic flip, in our test data, the multi-face removal operation can only handle more than ten tetrahedral elements at a time, which is more than basic flipping, but still limited. MFRC solves this problem. The MFRC algorithm is inspired by SPR (Small polyhedron re-connection)^20^. Although the target has not been changed, that is, seeking the best tetrahedralization of a polyhedron with a certain number of vertices and faces, we provide some novel ideas:

1. When selecting the excavation face, if there is a face with a dihedral angle greater than $180^{\circ }$, then this face is the first candidate. If there are multiple faces of this type, the face with the worst quality will be selected. Otherwise, directly select the worst-quality face.
2. Merge interior vertices and eliminate the worst interior elements.
3. Construct a memo for the quality and validity information of the known tetrahedral division.

The first and third items can ensure that the algorithm runs in an acceptable time, and the second item can more effectively eliminate the worst element and improve the overall quality.

## Related works

### Topology operation

Topology operations improve the shape of tetrahedral elements by changing the connection between vertices^14,17,19,21–25^. Common topology operations include 2-2 flip, 2-3 flip (3-2 flip), 4-4 flip. As shown in Fig.  1a–c. Joe presented the approach of using combinations of 2-2 flip, 3-2 flip, 4-4 flip to improve the quality of tetrahedral mesh^14^.

Edge removal is proposed by Briere^15^, which removes a single edge from mesh, along with all the tetrahedrons that contain it. Edge removal can be regarded as n-(2n-4) flip, whereas 3-2 flip and 4-4 flip are both simple forms of edge removal. As we can see in the middle of Fig.  1d. Multi-face removal is the inverse of edge removal^16^. Multi-face removal can be regarded as 2n-(n + 2) flip, 2-3 flip, and 4-4 flip are both simple forms of multi-face removal. Shewchuk proposed an optimization algorithm that can find the optimal edges and faces when performing edge removal and multi-face removal, respectively, so that the edge removal and multi-face removal operations can obtain the best results^26^. The algorithm uses a dynamic programming algorithm to find an optimal edge removal operation. Chen developed a new mesh local reconnection technique. This technique recursively calls a new method called shell transformation to optimize the tetrahedral mesh. The shell transformation can be regarded as an enhanced version of edge removal^19^. And thread-parallel can be combined with face and edge swapping and vertex insertion using in mesh improvement^21,27^. Multi-face retriangulation is illustrated in Fig. 1d. Multi-face retriangulation can be regarded as a combination of multi-face removal and edge removal. Multi-face retriangulation will not change the number of tetrahedron elements in the mesh and can be performed on boundary mesh^17^.

Different from the above-mentioned flip operation, the SPR algorithm proposed by Liu et al. aims to improve the mesh quality by finding the optimal tetrahedral division of the specified local mesh^20^. The input of the SPR algorithm is a polyhedral cavity containing low-quality elements. First, an initial face is selected, and then the optimal tetrahedral division of the polyhedron is found through exhaustive enumeration. Unlike the flip operation, the SPR algorithm can process a polyhedral cavity composed of 20–40 tetrahedral elements. Therefore, the SPR algorithm can obtain better results. Although the SPR algorithm can raise the local mesh quality to a higher level, it is difficult to apply SPR to specific mesh improvement methods. The reason for this situation is that the optimal tetrahedral division of polyhedrons is an NP-hard problem, and it is quite time-consuming to solve it through exhaustive methods. Although some optimization strategies are proposed to improve the performance of SPR^28^, the time performance in the actual application is still not completely satisfactory.

### Vertices insertion and contraction operation

Klingner et al. applied vertices insertion in mesh quality improvement which achieves an excellent improvement result but is time-consuming^18^. They improved the worst tetrahedral element in the mesh by inserting a vertex inside or on the boundary of the tetrahedral element. Edge contraction is a common operation that can simplify the mesh efficiently^29–32^. Klingner et al. used edge contraction to control the element size in tetrahedral mesh^18^. Wicke et al. proposed a dynamic mesh improvement scheme, which is aimed at tetrahedrons whose quality is below the minimum threshold. In this method, edge contraction is used to delete tetrahedral elements whose quality is degraded due to the existence of too short edges^33^. If vertices insertion and vertices removal are used to improve mesh quality, they can be effectively combined with other operations^18,27,34–36^.

### Smoothing

Smoothing can also be understood as geometric optimization, moves the vertices without changing the connection of vertices in the mesh. The most commonly used geometric optimization method is Laplacian smoothing^37^. Lei et al. proposed a GPU-accelerated parallel algorithm for Laplacian smoothing in three dimensions^38^. However, Laplacian smoothing cannot guarantee whether the mesh quality can be improved, especially in the case of three-dimensional mesh.

The more effective method is the optimized-based mesh quality improvement method^39–48^. Du uses a method based on CVT (centroidal Voronoi tessellations) for mesh smoothing^49,50^, Chen uses a method based on ODT (Optimal Delaunay Triangulations) for mesh smoothing^51^, and Zhong uses a particle-based method for mesh smoothing^52^. All these three methods (CVT-based method, ODT-based method, and particle-based method) define the global mesh energy and improve the mesh quality by minimizing the energy, so the worst element cannot be penalized, some local optimization methods (such as sliver perturber^53^ and sliver exuder^54^) can be combined to get better results. Freitag and Ollivier combined swapping and smoothing to improve tetrahedral mesh quality. They smoothed the inner vertices using a non-smooth optimization algorithm and obtained excellent results^22^. The boundary mesh has a large impact on the quality of the overall mesh, however, flipping operations have a limited improvement on boundary elements, and moving the position of boundary vertices might change the shape of the mesh model. Klingner used the non-smooth optimization algorithm for the inner vertices and implemented constrained smoothing for boundary vertices^18^. Franco et al. improved the tetrahedral mesh using moving mesh smoothing and dealt with curved boundary vertices using radial basis functions (RBFs)^55^.

## MFRC

### Main idea

The MFRC algorithm takes a cavity as an input which is formed by a group of tetrahedral elements containing the target element (low-quality tetrahedron). We choose a face as the starting point of the algorithm and consider all the vertices that may form a tetrahedron with the initial face. If the quality of the formed tetrahedron is lower than the worst quality of the original local mesh or the formed tetrahedron is invalid, the next face will be found and processed. Otherwise, if the found vertex is an interior vertex, insert a new element into the cavity and update the boundary of the cavity; If the vertex is on the boundary, insert a new element into the cavity, divide the original cavity into at most three new cavities through the boundary of the new tetrahedron, and then process the new cavities separately. Process each face recursively until the cavity is filled. After completing the entire search process, the optimal tetrahedral division of the cavity can be obtained.

**Figure 2:**
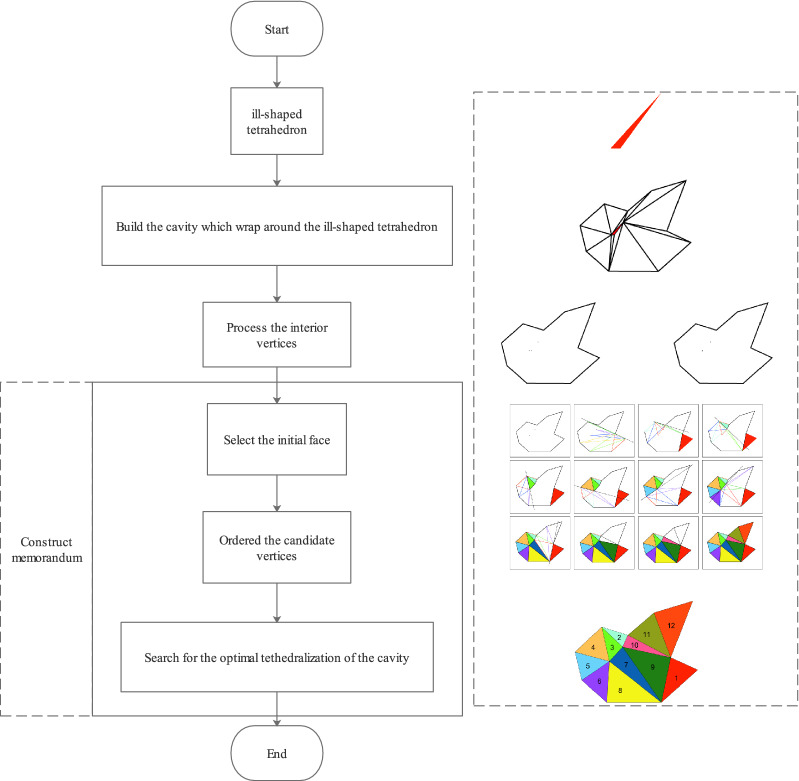
Flowchart of MFRC algorithm. The left side of the figure shows the implementation steps of the MFRC algorithm, and the right side is a simple diagram of the algorithm in a two-dimensional situation, where the numbers on the triangles indicate the order of processing.

Figure 2 shows the flowchart of the MFRC algorithm. The MFRC algorithm is executed in four steps. The first step is to initialize the cavity, including constructing the cavity by wrapping low-quality elements and processing the interior nodes of the cavity. The second step is to select the initial face. By selecting the initial face, on the one hand, it can be judged whether the sub-problem is meaningful, and on the other hand, it can classify the candidate vertices. The third step is to determine and sort the candidate vertices. The fourth step is to search for the optimal tetrahedral division of the cavity, and continuously update the cavity state in the process. In the process of searching for the optimal tetrahedral division, a memo is constructed to record the state of the algorithm to avoid repeated calculations.

### Build cavity

In the MFRC algorithm, the cavity that wraps the ill-shaped element must first be constructed, which can be a convex polyhedron or concave polyhedron. First, select the target low-quality element, then search for the neighboring of the target elements, and finally wrap the target element with the cavity.

According to the position of the low-quality element in the cavity, there are four situations: (1) One vertex is on the boundary of the cavity, and three vertices are inside the cavity. (2) Two are on the boundary of the cavity, and two vertices are inside the cavity. (3) Three vertices are on the boundary of the cavity, and one vertex is inside the cavity. (4) All four vertices are inside the cavity. According to the cavity construction principle of the MFRC algorithm, the distribution of the vertices in the cavity directly affects the results of the tetrahedral division of the cavity. The processing of interior vertices will be introduced in the next section. The number of vertices in the cavity directly affects the efficiency of the MFRC algorithm. Therefore, in actual application, to balance the mesh quality and execution efficiency, the MFRC algorithm will strictly control the number of vertices in the constructed cavity.

### Process the interior vertices

One of the keys to ensuring good element quality is to prevent unnecessary short sides. To eliminate the short edges or narrow elements in the cavity as much as possible, the vertices inside the cavity are merged in the MFRC algorithm.

When there are more than two interior vertices, the MFRC algorithm judges whether to merge them based on the distribution of the interior vertices. Since interior vertices will form new tetrahedrons with the boundary face of the cavity, the MFRC algorithm considers the relationship between the edges formed by the interior vertices and the boundary edges of the cavity to avoid generating tetrahedrons with a short edge as much as possible. Suppose cavity ${\varvec{C}}$ has m boundary edges and *n* interior vertices. The average length of the boundary edges is $lb_{avg}=\frac{1}{m}\sum _{i=1}^m{l_i}$ , *n* interior vertices can form $C_{n}^{2}$ edges, and the set of these edges is represented by *E*, if $\exists e\in E$, $\frac{lb_{avg}}{l_e}>3$, then merge the interior vertices.

When there are 3 vertices inside the cavity, consider the inner angle of the triangle built with the three vertices. If the maximum inner angle of the triangle is greater than $150^{\circ }$, the opposite side of this angle will be retained, and the third vertex would be merged into any other two vertices. When there are four vertices inside the cavity, consider the aspect ratio and solid angle of the tetrahedron. If the aspect ratio is greater than 3, shrink the shortest edge, and then deal with it in a three-vertex manner; If the solid angle of the tetrahedron is greater than 4.7124, delete the vertex where the current solid angle is located, and then deal with it in a three-vertex manner.

Since the cavity is constructed based on MFRC, the interior vertices that need to be processed are the vertices of the target low-quality elements. After processing, the low-quality elements in the cavity will be eliminated, and the short edge can be avoided to a certain extent.

### Select the initial face

**Figure 3:**
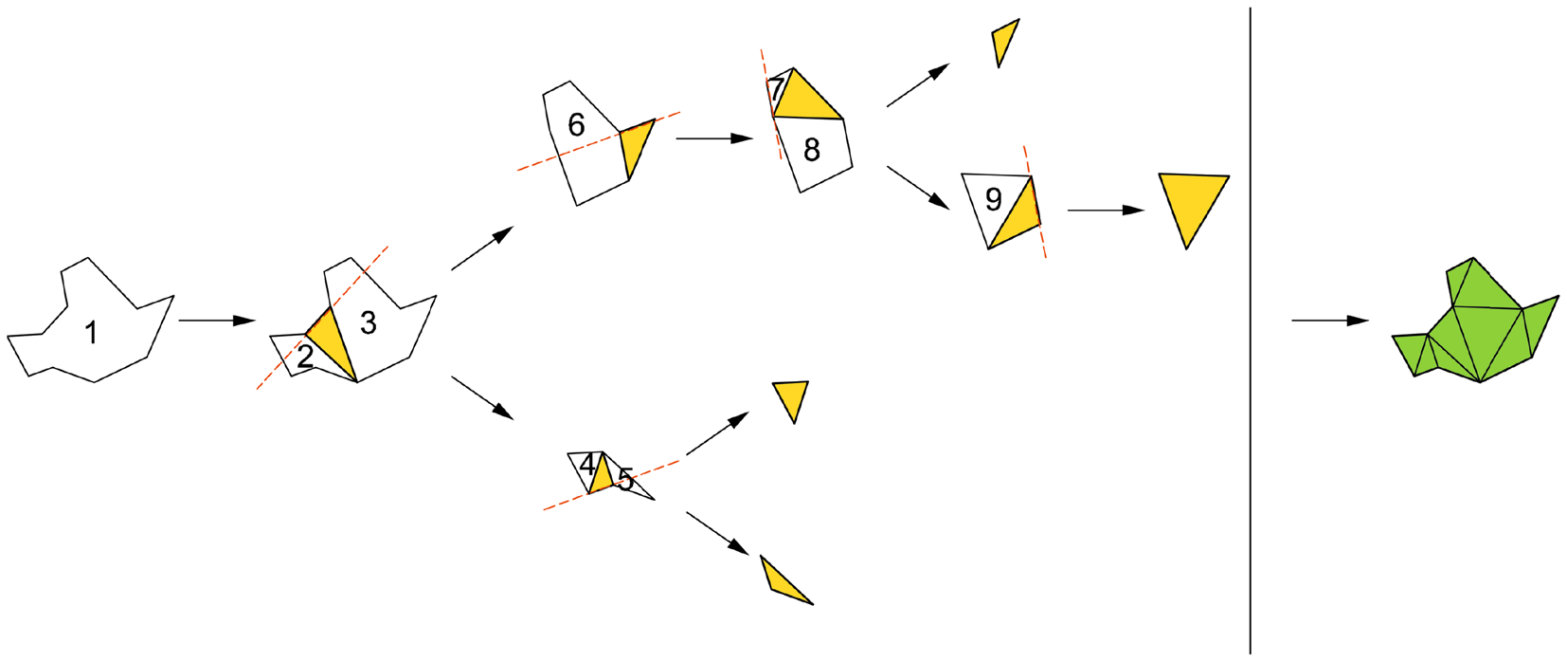
Two-dimensional MFRC algorithm. Each step will select a concave edge (the internal angle formed by the edge is greater than $180^{\circ }$), and each selection will filter out a part of the candidate vertices. The new triangle will be obtained to divide the whole to reduce the size, and the operation will be performed recursively until the entire area is filled.

When selecting the initial excavation face, in addition to the worst quality face, we also consider the concave face. If there is a face F on the boundary of the cavity, and the dihedral angle formed by F and the adjacent face is greater than $180^{\circ }$, the face F is called a concave face. In the MFRC algorithm, the concave face is preferentially selected as the initial face. The advantage of choosing a concave face is that the vertex of an effective tetrahedron can only appear on one side of the concave face, as shown in Fig. 3. This can intuitively reduce the number of candidate vertices involved in the selection, which can save time spent on tetrahedral validity detection and intersection detection.

In the MFRC algorithm, if the candidate vertex is the inner vertex of the cavity, first insert a new tetrahedron, and then use the faces of the new tetrahedron to update the boundary of the cavity; If the candidate vertex is located on the boundary of the cavity, insert a new tetrahedron first, then divide the original cavity into three sub-cavities at most, and use the new tetrahedron as the boundary. By solving the optimal tetrahedral division of the sub-cavity, the optimal tetrahedral division of the original cavity can be obtained. When the concave face is selected as the initial face, the original cavity can usually be directly decomposed into sub-cavities.

Choosing a concave face as the initial face does not mean that quality checks, validity checks, and intersection checks will not be performed when inserting a new tetrahedron, but that fewer checks will be performed. The vertex-face that has been checked will be saved in the memo so that it can be used directly in future checks.

### Order the candidate vertices

After selecting the initial face, it is necessary to select vertices that can form a tetrahedron with the initial face, and the search order of candidate vertices affects the convergence speed of the MFRC algorithm. The main consideration principles for candidate vertices sorting include (1) Consider whether the formed tetrahedron is valid, including checking the orientation of the tetrahedron and whether it intersects with other elements. (2) Consider the quality of the tetrahedron. Choosing a better tetrahedron is the original intention of the algorithm. (3) Consider the position of candidate vertices, adjacent boundary vertices > interior vertices > other vertices.

### Construct memorandum

In the process of finding the optimal tetrahedral division of the cavity, the quality information and effectiveness information of many tetrahedrons will be repeatedly calculated. Similarly, the tetrahedral division of some sub cavities will be calculated repeatedly. These repeated calculations will cause significant time consumption.

The MFRC algorithm constructs a tetrahedron-quality table. When it is necessary to calculate the quality of a tetrahedron, first check whether the quality of the tetrahedron exists in the table. If it does not exist, add the quality of the tetrahedron to this table after the calculation is completed. In the MFRC algorithm, some sub-cavities generated by the initial concave face needn’t be recalculated and will also be saved in the memo. Similarly, the MFRC algorithm constructs a digging-face-candidate-vertex table to detect whether a pair of digging-face and candidate-vertex constitute a valid tetrahedron.

### Termination condition

To ensure the efficiency and convergence of the algorithm, we introduce the termination conditions of the MFRC algorithm. If the sequence is empty or when the maximum number of iterations is reached or the mesh quality cannot be further improved, the algorithm terminates. The time consumption of the MFRC algorithm is determined by the number of boundaries of the cavity of the target low-quality tetrahedral element. To ensure the efficiency of the algorithm and the success rate of the algorithm, we tested multiple sets of cavities, including convex cavities and concave cavities. And when in use, we can set the maximum number of elements contained in the cavity. Algorithm 1 illustrates the implementation process of the MFRC algorithm. 
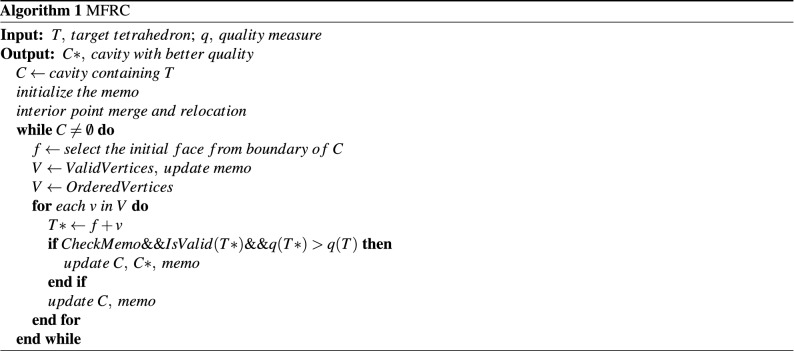


### MFRC algorithm deal with local mesh

**Figure 4:**
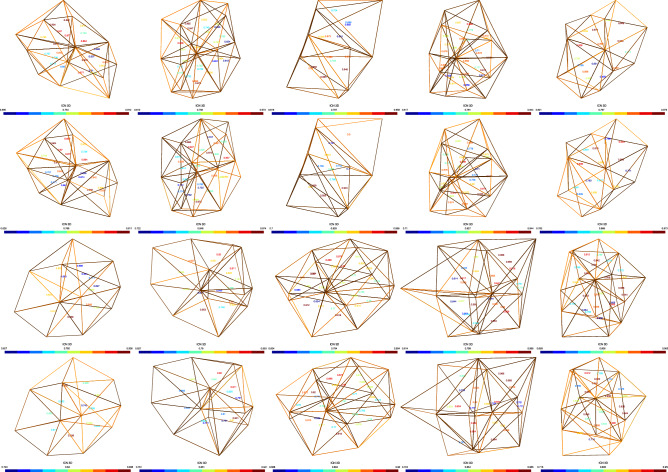
The result of the local mesh processing by the MFRC algorithm.

In order to prove the effectiveness of the MFRC algorithm, we tested the performance of the MFRC algorithm when dealing with local mesh. The meshes used for testing are derived from the actual mesh model and extracted from the mesh improvement process. And it is the result of the MFRC algorithm after the topology optimization fails (edge removal and multi-face removal). Figure 4 shows the element quality of mesh before and after MFRC, the value indicates the volume to length ratio of each tetrahedral element. It can be seen from Table 1 that the MFRC algorithm can effectively improve the quality of the local mesh.

**Table 1:** MFRC algorithm execution results.

case	#V	#F	^28^				Test1				Test2			
			#T	$\gamma _{min}$	$\gamma _{max}$	ITEs	#T	$\gamma _{min}$	$\gamma _{max}$	ITEs	#T	$\gamma _{min}$	$\gamma _{max}$	ITEs
1	14	24	17	0.531	1	30	18	0.530595	0.7698	27	19	0.349407	0.640032	34
2	15	26	20	0.416	0.77	36	20	0.336436	0.657267	30	22	0.370283	0.637798	29
3	16	28	22	0.416	0.657	130	23	0.272034	0.681418	37	23	0.242862	0.687	28
4	17	30	23	0.416	0.768	118	25	0.272034	0.681418	36	27	0.242862	0.683189	28
5	18	32	24	0.416	0.768	33	28	0.272034	0.681418	33	29	0.242862	0.721574	26
6	19	34	26	0.416	0.629	34	30	0.246114	0.811652	32	29	0.282828	0.748965	39
7	20	36	28	0.416	0.629	38	32	0.272034	0.871645	38	31	0.282828	0.721842	31
8	21	38	30	0.416	0.629	40	34	0.272034	0.785062	35	33	0.287991	0.676349	35
9	22	40	32	0.416	0.521	41	36	0.278843	0.636575	40	37	0.287991	0.583465	38
10	23	42	33	0.416	0.794	42	38	0.278843	0.871645	37	37	0.27237	0.581649	25
11	24	44	34	0.416	0.794	42	39	0.272034	0.785062	41	42	0.269072	0.557604	34
12	25	46	35	0.416	0.794	41	42	0.272034	0.75403	40	44	0.269072	0.737899	45
13	26	48	36	0.416	0.794	36	44	0.278843	0.647611	41	45	0.284921	0.669754	39
14	27	50	38	0.266	0.794	43	48	0.278843	0.700625	37	47	0.284921	0.681691	42
15	28	52	40	0.266	0.794	50	51	0.272034	0.583822	39	48	0.275795	0.795804	37
16	29	54	42	0.266	0.794	58	52	0.272034	0.746506	46	51	0.275795	0.665748	33
17	30	56	44	0.266	0.794	66	53	0.278843	0.805621	45	56	0.264591	0.942604	36
18	31	58	46	0.266	0.794	74	56	0.278843	0.701426	43	57	0.27003	0.812548	42
19	32	60	48	0.266	0.794	83	57	0.272034	0.632659	54	58	0.265605	0.856217	55

#V is the number of vertices, #F is the number of faces, #T is the number of elements, ITEs is the number of iterations.

Reference^28^ uses the $\gamma$ coefficient to measure the quality of the tetrahedron, which can be expressed as $\gamma =72\sqrt{3}\frac{V}{\left( \sum _{1\leqslant i\leqslant j\leqslant 4}{l_{ij}^{2}} \right) ^{1.5}}$, where *V* represents the volume of the tetrahedron, and $l_{ij}$ represents the edge length. The polyhedron used for testing in reference^28^ is a special convex structure; all nodes in the polyhedron are co-spherical, and a total of 19 test meshes are constructed. The first example has 14 nodes and 24 faces. The other examples were created from the first example by inserting points by points. The last example has 32 nodes and 60 faces.

In this article, we constructed two sets of data. The first one is to take points on the sphere relatively uniformly, generate a surface grid based on these points, and then insert point by point. The second one randomly picks points on the sphere, generates a surface grid based on these points, and then inserts point by point. However, since there is no mention of the way or position of node insertion in reference^28^, although we give the quality and the number of elements, we only consider the number of iterations. The test results are shown in the table below. From the data in Table 1, the MFRC method can complete the tetrahedral division of the cavity with fewer iterations in most examples.

## Implementation

The purpose of this research is to develop an efficient tetrahedral mesh improvement method. To obtain better improvement results, the proposed method combines mesh smoothing, edge removal, multi-face removal and vertex insertion/deletion. The volumelength measure is numerically and can punish all types of low-quality tetrahedrons, so the volume–length measure is used in this study. Since the overall quality of the mesh depends on the quality of the worst element, the mesh improvement operation is only performed on the low-quality elements, with clear goals and saving time. The boundary elements in the mesh have a great influence on the quality of the mesh, but in many practical applications, the improved mesh needs to be consistent with the original geometric model. Therefore, in this method, it is not allowed to change the boundary elements. The entire mesh improvement process adopts the hill climbing. When performing each local improvement, the optimized mesh quality must be higher than the initial mesh quality, otherwise no operation is allowed. This method is based on the MFRC algorithm and combined with the following operations, including:

*Mesh smoothing* In this method, Laplacian smoothing, and optimization-based smoothing are combined. First, perform Laplacian smoothing on the target mesh, if successful, continue smoothing, otherwise perform optimization-based smoothing. In each smoothing process, each non-smooth node is processed only once^25^. If the mesh quality (including the worst quality and average quality) cannot be further improved, the smoothing process ends.

*Edge removal, multi-face removal* For edge removal, the dynamic programming algorithm proposed by Klincsek^52^ is used to solve the optimal triangulation problem of polygons, so as to realize the optimal edge removal operation. For multi-face removal, the algorithm proposed by Shewchuk^29^ is used to find the optimal face that allows to be deleted.

*Vertex insertion, vertex deletion* For vertex insertion, for a tetrahedron whose edge length ratio is too large, insert a vertex on the longest edge to split the tetrahedron. If the quality of the newly generated tetrahedrons is better than that of the original tetrahedrons, the insertion vertex and the new tetrahedrons are retained, otherwise the vertex is deleted and the tetrahedron is not allowed to split. For vertex deletion, on the one hand, it is used to process the interior vertices of the MFRC algorithm, and on the other hand, it tries to improve the mesh quality through edge contraction operations.

In order to eliminate too long or too short edges in low-quality elements, we perform size control on the mesh^53^ with vertex insertion and vertex deletion. After the size control, we perform smoothing to overall mesh, using Laplacian smoothing combined with optimization-based smoothing. Then the main loop is executed, and the low-quality elements list is updated first at the beginning of each loop. Perform smoothing on the elements in the list. If smoothing fails, perform the topology process. If neither smoothing nor topology optimization can improve the quality of the target element, the MFRC algorithm is called. Note that after each local reconstruction (topology optimization, MFRC and vertex insertion, vertex deletion), the local mesh is smoothed. When none of the above operations can improve the mesh or reach the iteration threshold or the mesh upgrade to the target quality, the iteration improvement ends. Algorithm 2 presented the whole mesh improvement method. 
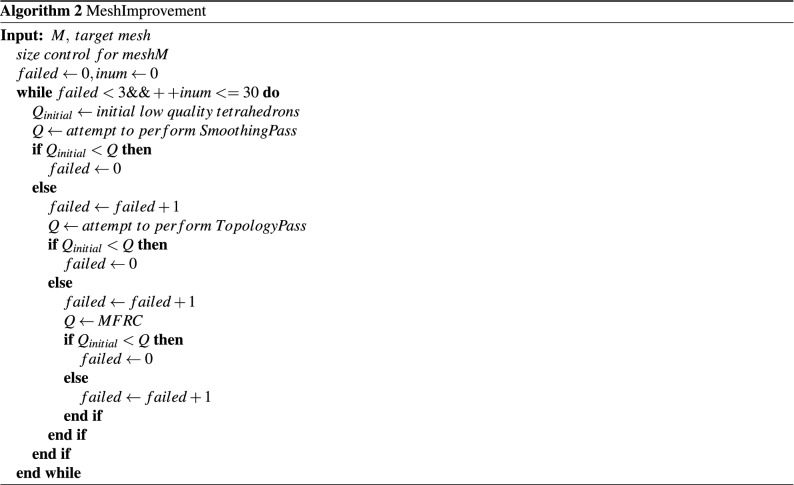


## Results discussion and analysis

### Mesh quality measure

To improve the quality of the tetrahedral mesh, the quality measure of the tetrahedral mesh should be determined. The following are some commonly used quality metrics of tetrahedron meshes: (1) dihedral angle. In the finite element method, large dihedral angles hurt the accuracy of simulation and small dihedral angles affect stiffness matrix conditioning^8^. (2) volume–length ratio. Dannelongue et al. proposed to use volume and the average edge length to characterize a tetrahedron^56^. V.N. et al. extended the work of Dannelongue and presented the volume–length measure. Volume–length measure can be calculated as $q=V /\left( l_{r m s}^{3}\right)$, where *q* is the quality value, *V* is the volume of a tetrahedron and $l_{rms}$ is the root-mean-squared edge length of a tetrahedron^57^.

### Results and analysis

In this section, we test our proposed mesh improvement method on some mesh models to verify the effectiveness and compare the results with Mmg^58^, Stellar^18^ and mesh optimization methods provided in the Computational Geometry Algorithm Library (CGAL). CGAL provides four mesh optimization methods, including two global methods: Lloyd smoother^49^ and ODT smoother^51^ and two local methods: Vertex Perturb^59^ and Sliver Exuder^54^. The global method first defines the energy function of the mesh quality, and minimizes the energy function by relocating all vertices in the mesh, thereby achieving the improvement of the mesh quality. The local method gives priority to the worst-quality element, the so-called sliver. Vertex perturb starts from the worst tetrahedron, tries to add a small disturbance to each vertex of the target tetrahedron and updates the connectivity of the vertices to eliminate sliver. Sliver exuder converts triangulation into weighted triangulation and eliminates sliver by adjusting the distribution of vertex weights. The numerical tests were conducted on a PC (CPU: 3.0GHz, Memory: 8GB), Gmsh (http://www.gmsh.info/^60^) and OriginPro Learning Edition (https://www.originlab.com/OriginProLearning.aspx) are used for mesh rendering and scientific drawing. As far as we know, Stellar is the best open-source mesh improvement method. The purpose of Stellar is to optimize the worst tetrahedral element as much as possible, and efficiency is a secondary consideration. Mmg is an open-source software for remeshing 2D and 3D surfaces and volumes, and it is still actively updated. Moreover, Mmg provides an effective tetrahedral mesh improvement function. Stellar + MFRC means the combination of the Stellar method and the MFRC algorithm, that is, using the MFRC algorithm replaces the topology processing part of the Stellar method.

**Figure 5:**
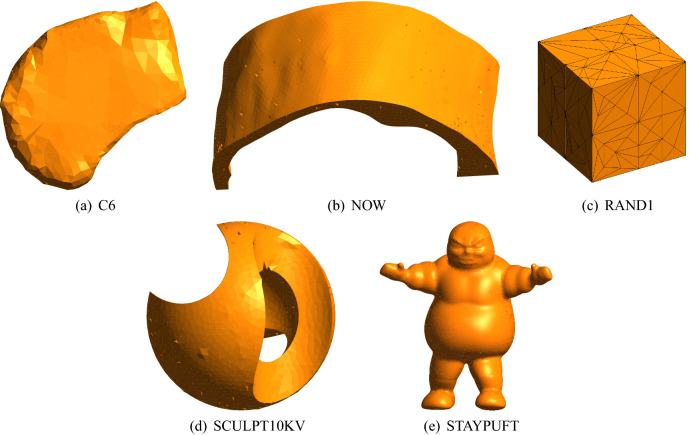
Example meshes for comparison. The meshes are rendered using the open source software Gmsh.

In order to compare the effects of the MFRC algorithm and other methods, we compared the dihedral angle, volume–length ratio, distribution range of the dihedral angle and running time of different methods. Examples of multiple mesh models used in this article are shown in Fig. 5.

**Table 2:** Quality statistics of mesh model, include min dihedral $\theta _{min}$, max dihedral $\theta _{max}$, min volume–length ratio $vl_{min}$, max volume–length ratio $vl_{max}$, number of elements #tet, running time.

Model	Method	$\theta _{\min }/\theta _{\max }$	$vl_{min}/vl_{max}$	#$<10^{\circ }$	#$<20^{\circ }$	#$<30^{\circ }$	#$<40^{\circ }$	#tet	Time (s)
C6	Init	2.88/172.84	0.131/0.997	2	22	250	1810	6946	–
	Lloyd	1.02/177.02	0.02/0.997	77	240	498	1004	2354	4.015
	ODT	1.15/173.35	0.014/0.996	44	160	420	1001	2324	0.534
	Exude	1.41/167.00	0.031/0.992	15	156	715	2188	6703	1.01
	Perturb	1.73/176.03	0.024/0.993	228	678	1294	2711	7009	2.457
	Mmg	2E−5/179.99	1.32E−9/0.972	384,549	675,299	922,455	1,129,616	459,905	38.7
	Stellar	7.35/142.54	0.151/0.988	2	11	81	2276	8093	183.64
	MFRC	7.35/146.18	0.151/0.991	2	11	182	2071	8060	5.94
	Lloyd + Exude	2.27/169.26	0.013/0.998	31	85	221	646	2292	3.21
	Lloyd + Perturb	4.86/171.36	0.012/0.997	51	197	458	960	2342	10.539
	MFRC + Lloyd	7.35/147.73	0.09/0.994	3	10	75	1101	6822	13.08
	ODT + Exude	2.46/164.12	0.037/0.997	21	72	231	725	2277	1.28
	ODT + Perturb	6.27/164.07	0.042/0.997	14	109	337	884	2302	4.6
	MFRC + ODT	7.35/150.74	0.117/0.994	7	19	51	907	7198	7.68
	Stellar + Exude	7.35/148.82	0.103/0.991	14	46	145	1723	7123	20.07
	Stellar + Perturb	7.35/147.51	0.125/0.992	14	48	133	1575	6184	25.62
	Stellar + MFRC	7.35/142.33	0.151/0.992	2	13	69	2008	8026	28.312

**Table 3:** Quality statistics of mesh model, include min dihedral $\theta _{min}$, max dihedral $\theta _{max}$, min volume–length ratio $vl_{min}$, max volume–length ratio $vl_{max}$, number of elements #tet, running time.

Model	Method	$\theta _{\min }/\theta _{\max }$	$vl_{min}/vl_{max}$	#$<10^{\circ }$	#$<20^{\circ }$	#$<30^{\circ }$	#$<40^{\circ }$	#tet	Time (s)
NOW	Init	1.13/175.94	0.085/0.998	6	563	4362	8345	19902	–
	Lloyd	0.69/178.88	0.012/0.999	405	1331	3057	7451	25647	24.28
	ODT	2.04/176.77	0.035/0.999	57	287	1192	5216	25127	8.58
	Exude	11.15/160.47	0.141/0.999	0	192	1804	6081	20166	4.388
	Perturb	11.97/161.93	0.141/0.999	0	1000	2978	6977	20630	3.392
	Mmg	1E−5/179.99	6.94E−8/0.95	338,335	595,944	814,254	1,001,834	435,519	163.39
	Stellar	35.81/142.23	0.263/0.999	0	0	0	1555	25247	707.18
	MFRC	32.85/139.86	0.45/0.997	0	0	0	3611	19821	38.44
	Lloyd + Exude	8.05/167.05	0.137/0.999	3	54	888	4606	25160	24.78
	Lloyd + Perturb	24.24/144.14	0.356/0.998	0	0	669	4429	25137	24.32
	MFRC + Lloyd	33.36/128.43	0.482/0.999	0	0	0	2158	21526	39.81
	ODT + Exude	14.90/152.77	0.261/0.999	0	21	561	4173	24935	11.7
	ODT + Perturb	25.93/145.83	0.411/0.999	0	0	381	3921	24893	15.61
	MFRC + ODT	34.63/133.87	0.486/0.997	0	0	0	2721	22072	28.1
	Stellar + Exude	32.29/131.49	0.47/0.995	0	0	0	3368	22814	735.82
	Stellar + Perturb	32.31/131.69	0.47/0.997	0	0	0	3427	22572	685.98
	Stellar + MFRC	37.85/126.86	0.557/0.999	0	0	0	3063	27182	847.53

**Table 4:** Quality statistics of mesh model, include min dihedral $\theta _{min}$, max dihedral $\theta _{max}$, min volume–length ratio $vl_{min}$, max volume–length ratio $vl_{max}$, number of elements #tet, running time.

Model	Method	$\theta _{\min }/\theta _{\max }$	$vl_{min}/vl_{max}$	#$<10^{\circ }$	#$<20^{\circ }$	#$<30^{\circ }$	#$<40^{\circ }$	#tet	Time (s)
RAND1	Init	0.32/178.96	0.0035/0.978	1094	3664	6575	9786	5104	–
	Lloyd	2.34/175.45	0.041/0.997	129	420	1000	2344	7242	10.62
	ODT	6.69/166.39	0.124/0.998	3	57	272	1504	7071	2.35
	Exude	11.15/160.20	0.192/0.998	0	34	305	1600	5507	0.601
	Perturb	12.23/162.25	0.204/0.998	0	268	746	1953	5672	0.173
	Mmg	0.0017/179.99	3E−5/0.977	3238	6406	9202	11796	5464	2.21
	Stellar	40.65/137.92	0.414/0.981	0	0	0	0	1283	111.71
	MFRC	37.78/140.63	0.435/0.982	0	0	0	403	2250	3.38
	Lloyd + Exude	13.35/159.01	0.233/0.997	0	13	243	1375	7076	11.64
	Lloyd + Perturb	27.09/140.08	0.371/0.997	0	0	81	1260	7056	17.06
	MFRC + Lloyd	35.08/118.30	0.61/0.993	0	0	0	218	5641	15.9
	ODT + Exude	15.95/150.35	0.294/0.997	0	7	92	1111	6998	5.04
	ODT + Perturb	31.05/134.27	0.463/0.998	0	0	0	1146	6998	33.146
	MFRC + ODT	35.83/117.03	0.57/0.997	0	0	0	208	6278	14.36
	Stellar + Exude	33.54/116.7	0.54/0.995	0	0	0	312	5744	319.87
	Stellar + Perturb	35.51/115.53	0.62/0.995	0	0	0	177	4880	346.27
	Stellar + MFRC	41.12/117.64	0.693/0.987	0	0	0	0	1075	377.18

**Table 5:** Quality statistics of mesh model, include min dihedral $\theta _{min}$, max dihedral $\theta _{max}$, min volume–length ratio $vl_{min}$, max volume–length ratio $vl_{max}$, number of elements #tet, running time.

Model	Method	$\theta _{\min }/\theta _{\max }$	$vl_{min}/vl_{max}$	#$<10^{\circ }$	#$<20^{\circ }$	#$<30^{\circ }$	#$<40^{\circ }$	#tet	Time (s)
SCULP T10KV	Init	11.45/161.74	0.207/0.999		170	2047	7431	50391	–
	Lloyd	0.26/179.51	0.004/0.999	577	2134	4752	10960	39427	75.12
	ODT	1.79/177.13	0.032/0.999	39	356	1707	7506	38752	12.678
	Exude	6.23/165.02	0.095/0.998	12	616	4561	14646	47542	6.757
	Perturb	12.00/163.29	0.076/0.998	0	2875	8040	17332	48866	2.842
	Mmg	2E−4/179.99	3.56e−6/0.963	40374	74612	102005	125039	51966	2.63
	Stellar	32.92/125.75	0.514/0.999	0	0	0	1989	45994	194.93
	MFRC	34.41/131.84	0.455/0.998	0	0	0	1752	52580	6.179
	Lloyd + Exude	3.5/160.41	0.008/0.999	6	129	1228	5208	38676	77.22
	Lloyd + Perturb	5.14/170.82	0.082/0.999	320	1539	2605	6196	39358	76.18
	MFRC + Lloyd	35.21/138.89	0.494/0.996	0	0	0	1001	35571	22.39
	ODT + Exude	4.55/160.48	0.099/0.999	5	39	665	5754	38431	20.705
	ODT + Perturb	7.25/165.22	0.118/0.999	10	369	1678	7476	38744	21.802
	MFRC + ODT	32.71/136.37	0.465/0.999	0	0	0	1048	37717	21.35
	Stellar + Exude	31.63/135.92	0.482/0.996	0	0	0	8756	29325	137.73
	Stellar + Perturb	32.67/132.09	0.514/0.997	0	0	0	8170	27789	151.48
	Stellar + MFRC	36.37/123.98	0.586/0.999	0	0	0	2144	45892	142.17

**Table 6:** Quality statistics of mesh model, include min dihedral $\theta _{min}$, max dihedral $\theta _{max}$, min volume–length ratio $vl_{min}$, max volume–length ratio $vl_{max}$, number of elements #tet, running time.

Model	Method	$\theta _{\min }/\theta _{\max }$	$vl_{min}/vl_{max}$	#$<10^{\circ }$	#$<20^{\circ }$	#$<30^{\circ }$	#$<40^{\circ }$	#tet	Time (s)
STAY PUFT	Init	1.14/177.22	0.024/0.998	645	3680	12059	34913	102392	–
	Lloyd	0.61/179.09	0.0102/0.999	1210	4853	11,738	27,530	122,265	69.65
	ODT	1.63/177.48	0.027/0.999	89	1082	4809	19,954	120,696	24.57
	Exude	6.02/170.66	0.104/0.999	6	993	10,003	34,961	122,431	16.53
	Perturb	12.00/162.89	0.174/0.999	0	7402	19,792	42,694	126,477	11.205
	Mmg	1E−6/179.99	2.5E−10/0.995	4,136,984	7,013,348	9,405,191	11,431,100	4,829,617	604.65
	Stellar	23.4/129.46	0.48/0.999	0	0	31	19,175	127,893	2257.82
	MFRC	26.34/144.03	0.411/0.995	0	0	492	13,989	103,200	152.37
	Lloyd + Exude	14.99/157.09	0.252/0.999	3	73	2972	15,719	120,250	71.87
	Lloyd + Perturb	23.98/146.6	0.385/0.999	0	0	2963	16,094	120,332	75.03
	MFRC + Lloyd	27.94/139.38	0.465/0.999	0	0	2	4051	99,068	197.41
	ODT + Exude	8.66/162.86	0.159/0.999	1	51	2066	15,945	120,019	24.68
	ODT + Perturb	24/146.67	0.48/0.999	0	0	0	10,200	119,083	121.89
	MFRC + ODT	25.53/131.92	0.538/0.998	0	0	0	4221	108,539	187.67
	Stellar + Exude	20.89/124.29	0.435/0.998	0	0	6	8855	102,677	2022.81
	Stellar + Perturb	20.89/128.33	0.434/0.998	0	0	17	9809	92,921	2358.23
	Stellar + MFRC	33.27/134.26	0.483/0.998	0	0	0	18,689	127,440	3127.03

**Figure 6:**
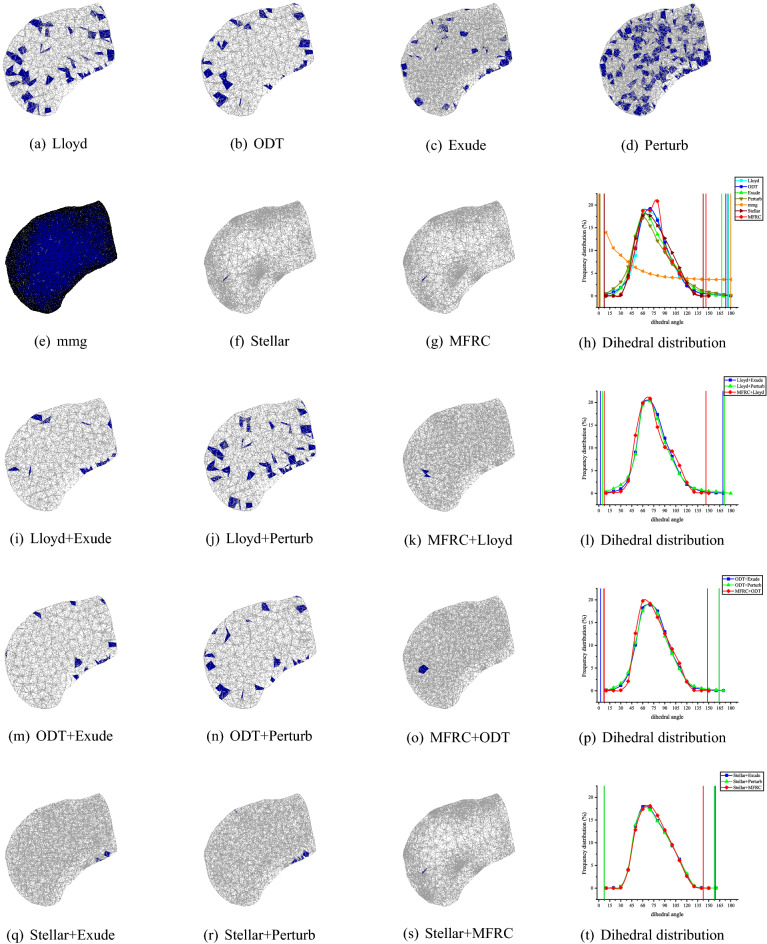
C6 volume meshing (2416 vertices) with anisotropic variation in a single direction. The blue ones are tetrahedron with smallest volume–length ratio less than 0.3. The meshes are rendered using the open source software Gmsh, and the Line + Symbol plot is drawn by OriginPro Learning Edition.

**Figure 7:**
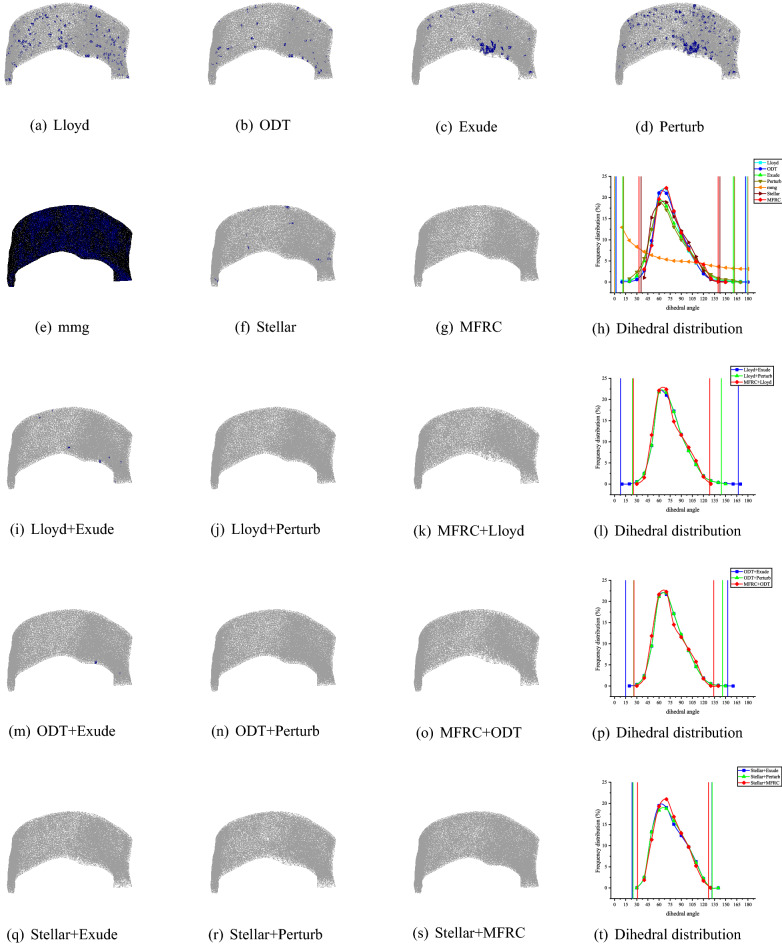
NOW volume meshing (8000 vertices) with anisotropic variation in a single direction. The blue ones are tetrahedron with smallest volume–length ratio less than 0.3. The meshes are rendered using the open source software Gmsh, and the Line + Symbol plot is drawn by OriginPro Learning Edition.

**Figure 8:**
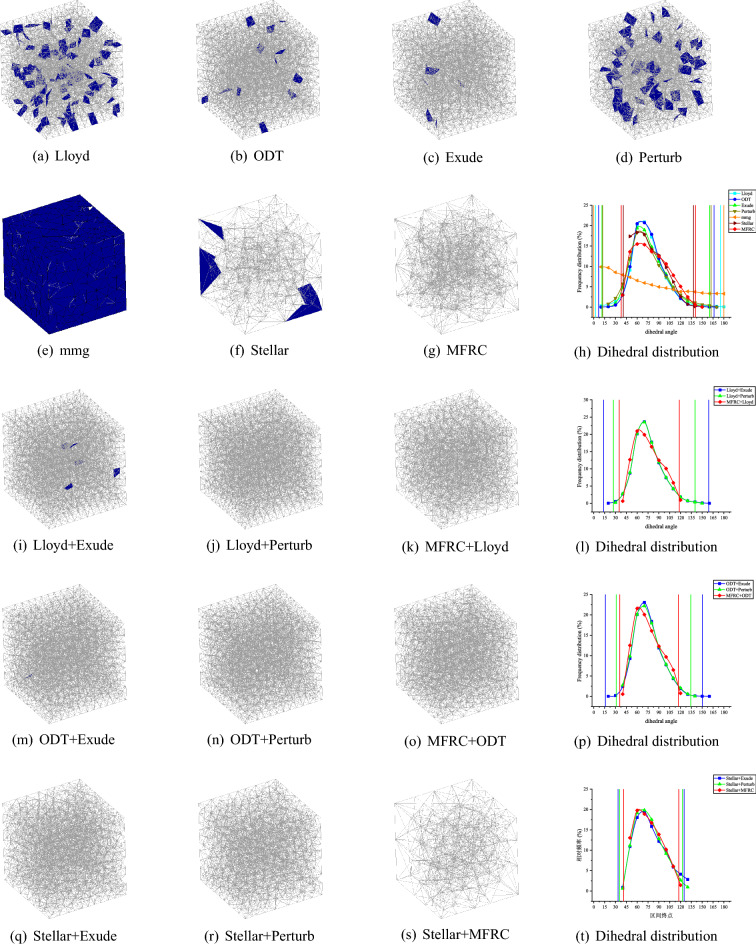
RAND1 volume meshing (1083 vertices) with anisotropic variation in a single direction. The blue ones are tetrahedron with smallest volume–length ratio less than 0.3. The meshes are rendered using the open source software Gmsh, and the Line + Symbol plot is drawn by OriginPro Learning Edition.

**Figure 9:**
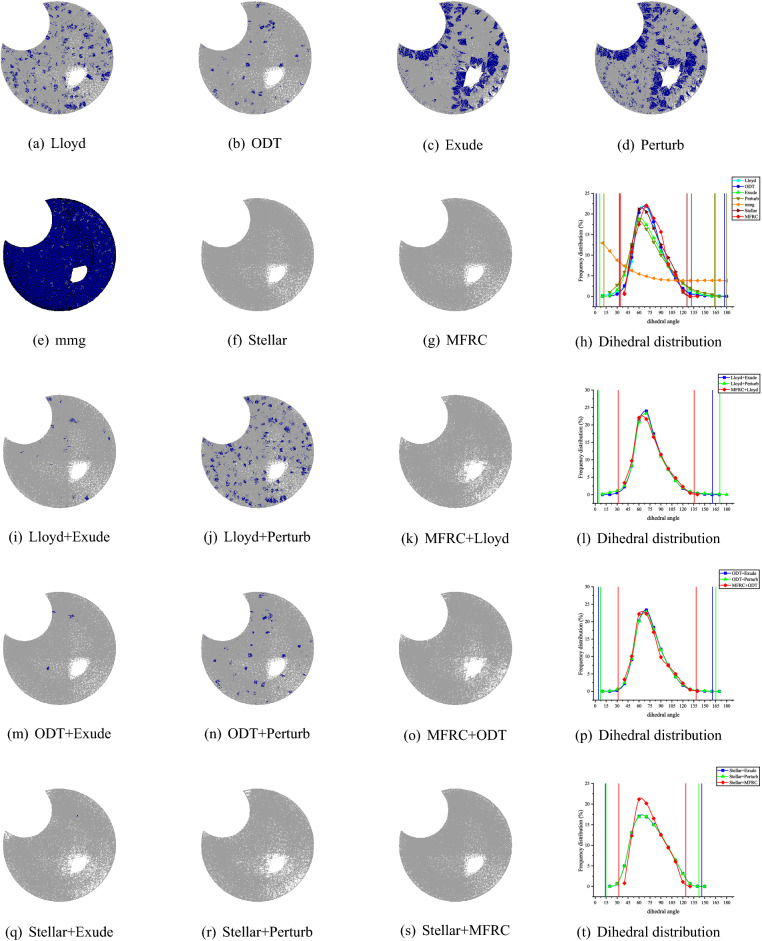
SCULPT10KV volume meshing (10,000 vertices) with anisotropic variation in a single direction. The blue ones are tetrahedron with smallest volume–length ratio less than 0.3. The meshes are rendered using the open source software Gmsh, and the Line + Symbol plot is drawn by OriginPro Learning Edition.

**Figure 10:**
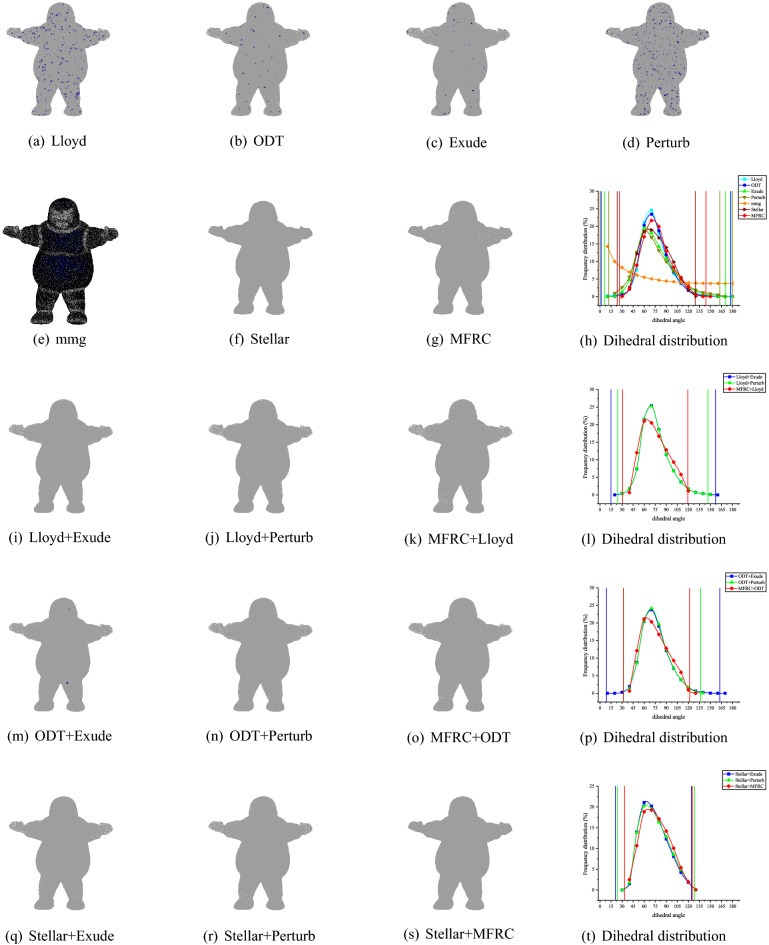
SCULPT10KV volume meshing (27,000 vertices) with anisotropic variation in a single direction. The blue ones are tetrahedron with smallest volume–length ratio less than 0.3. The meshes are rendered using the open source software Gmsh, and the Line + Symbol plot is drawn by OriginPro Learning Edition.

Consider the dihedral angle and the volume–length ratio of the mesh model. Tables 2, 3, 4, 5, 6 shows that the MFRC method can ensure that the maximum dihedral angle is less than 146.18°, and the minimum dihedral angle is greater than 26.34° on most mesh models. The exception is C6, the least dihedral angle is 7.35°, because the least dihedral angle appears on the surface of the mesh. In our experiment, all mesh optimization methods are set to ensure that the surface shape of the mesh model cannot be damaged. Considering most situations, MFRC performs slightly worse than the Stellar method, better results than Mmg and the optimization methods provided by CGAL. Similarly, considering the volume–length ratio, the MFRC algorithm can always effectively improve the volume–length ratio of the meshes. Figures 6, 7, 8, 9, 10 shows the mesh results processed by different methods, showing that the volume–length ratio of the element is lower than 0.3. Except for a few cases (C6), the volume–length ratio obtained by the MFRC algorithm is higher than 0.3. Combined with the observation Tables 2, 3, 4, 5, 6, it is better than the optimization methods provided by MMG and CGAL and is comparable to the effect of the Stellar algorithm.

**Figure 11:**
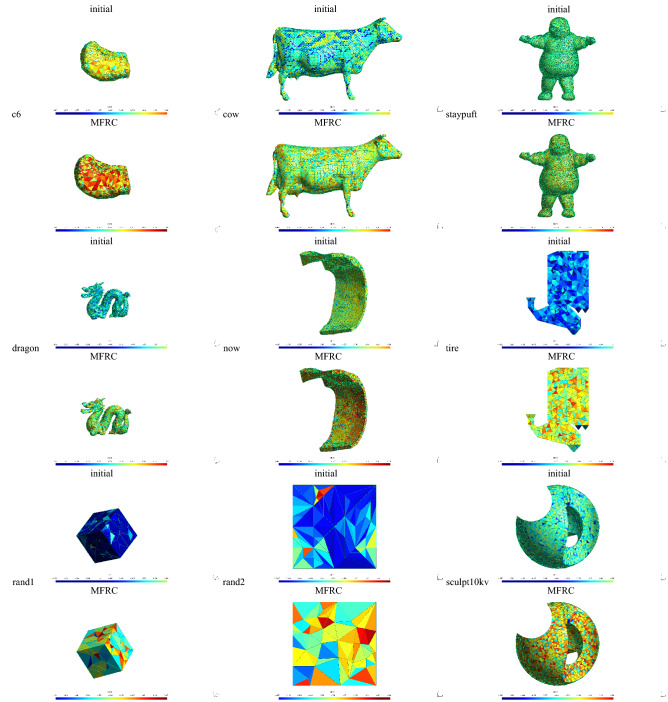
Meshes before and after MFRC improvement.

Consider mesh improvement processing time. The processing time of mesh improvement depends on the mesh scale on the one hand, and on the initial quality of the mesh on the other hand. According to Tables 2, 3, 4, 5, 6, compared with the optimization algorithms provided by Mmg and CGAL, although the running time of the MFRC algorithm is slightly lower than the above algorithm in some cases. But overall, the running time of the MFRC algorithm is still acceptable, especially compared to the Stellar method. The Stellar method is time-consuming, and the aggressive vertex insertion operation takes up most of the running time. In the MFRC method, vertex insertion is also used, but it is limited to inserting vertices on the edge, and vertex insertion does not consume much time. It should be noted that in the MFRC method, the main factor affecting the processing time is the size of the cavity, specifically the number of nodes that make up the cavity. In order to balance the processing time and mesh processing quality, we limit the number of nodes contained in the cavity at run-time. In actual operation, users can choose the size of the cavity according to their needs, but it should be noted that too large a cavity will lead to a long running time. Figure 11 shows the meshes processed using the MFRC method. The change in color tone from cold to warm indicates an improvement in the quality of the mesh.

In addition to the comparison of single methods, the paper also compares the combined methods. Combine Lloyd, ODT, and Stellar with Exude, Perturb, and MFRC respectively. It can be seen from the data in Tables 2, 3, 4, 5, 6 that the combined method improves the mesh quality more significantly than the single method. More specifically, when Lloyd, ODT and Stellar are used in combination with MFRC, they have achieved excellent mesh optimization effects in test cases, and the optimization effect used in combination with Perturb is the second best. Although the optimization effect obtained by combining Exude with Lloyd, ODT, and Stellar is better than that of the single method, it is slightly worse than the combined method of MFRC. This is also consistent with the optimization results obtained using a single method (MFRC, perturb, exude), that is, MFRC has the best effect, followed by Perturb, and Exude is slightly worse than MFRC and perturb.

Through comprehensive consideration of the worst quality of the mesh, the distribution of mesh quality, and the running time of mesh improvement, it can be concluded that:

The MFRC method proposed in this paper is a local mesh optimization method. The difference between MFRC and vertex perturb and sliver exuder is that the MFRC is aimed at a cavity containing the worst element. The purpose is to find a better quality triangulation, and it does not strictly require the cavity to have Delaunay characteristic. The reason for ensuring the advantages of MFRC is that, on the one hand, the tetrahedron is directly constructed on the basis of the existing cavity. In the actual operation, the cavity often contains dozens of elements, and the operation in a larger range leads to better local solution, thereby improving the mesh quality to a higher level^19,20^. Taking into account the efficiency issue, the validity and optimality of the elements are used to prune, and the memory search is used to speed up the algorithm. On the other hand, when there are vertices inside the cavity, the interior vertices are directly repositioned or the vertices are merged first and then re-adjusted the position of vertices. When local operations such as edge removal and multi-face removal fail, the adjustment of interior vertices by the MFRC can eliminate the worst element, and the reconstruction of the cavity can further improve the quality of the local mesh.

The mesh improvement method based on the MFRC algorithm can not only improve the quality of the input mesh to a higher level in a short running time (considering the worst quality and quality distribution, but it is also very close to the improvement effect of the Stellar method). Under the default command, Mmg will insert a large number of nodes into the input mesh, causing the number of elements to become huge. The Stellar method can undoubtedly effectively improve the quality of the input mesh, but the running time limits the application of Stellar, and Stellar changes the number of elements more than the MFRC method. Lloyd and ODT are global methods, and better results can be obtained when combined with local optimization methods. In this case, the MFRC algorithm still got better results. Therefore, after considering the cost and benefit, for single method, the MFRC method is better than the tetrahedral mesh optimization method of Mmg, Stellar and methods provided by CGAL. Considering the combination of MFRC and other global methods, although the running time is slightly higher than the other combination methods, the lowest element quality and dihedral angle distribution both get better results. For the three combination methods of MFRC in the paper, MFRC+Stellar provides better optimization results, but considering the time consumption, in practical applications, MFRC+ODT or MFRC+Lloyd may be a better choice.

## Conclusion

This paper proposes a local mesh reconnection algorithm, MFRC, which can obtain the optimal tetrahedral division of the local mesh. MFRC considers the concave face when selecting the initial face, directly filters out the vertices that do not meet the requirements and builds a memo to record the calculated element quality information, effectiveness information and effective tetrahedral division. MFRC reasonably merges interior vertices to obtain higher-quality tetrahedral division. MFRC allows the mesh to be changed in a wider range, so when the routine topological operation fails, MFRC can often achieve better results. The experimental results prove that the MFRC method can effectively improve mesh quality and achieve a good balance between mesh quality and running time.

Many mesh generation methods will create the worst element on the boundary. Due to the importance of boundary geometric features, the quality optimization of boundary elements is a very challenging research topic. Therefore, many researchers have conducted extensive and in-depth research on this, such as inserting vertices in boundary surfaces, constrained smoothing, and some research on feature preserving. Although the MFRC method does not focus on the boundary elements, it also improves the quality of the boundary elements to a certain extent. In the future, we will apply the MFRC method to the virtual surgery training system, and the geometric characteristics of the tissue (shape, size, roughness, etc.) play an important role in disease diagnosis and treatment. Therefore, in the current research, in order to retain the complete geometric features of the model, we did not consider complex processing of the mesh surface. The optimization of surface elements will be part of our future research work.

## Data Availability

The data used to support the findings of this study are available from the corresponding author upon request.
